# Growth of low temperature silicon nano-structures for electronic and electrical energy generation applications

**DOI:** 10.1186/1556-276X-8-83

**Published:** 2013-02-15

**Authors:** Nare Gabrielyan, Konstantina Saranti, Krishna Nama Manjunatha, Shashi Paul

**Affiliations:** 1 Emerging Technologies Research Centre, De Montfort University, Hawthorn Building, The Gateway, Leicester LE1 9BH, UK

**Keywords:** Silicon nano-wire, Nano-tree, Gallium, PECVD, Solar cell, Schottky diode, Bistable memory

## Abstract

This paper represents the lowest growth temperature for silicon nano-wires (SiNWs) via a vapour-liquid–solid method, which has ever been reported in the literature. The nano-wires were grown using plasma-enhanced chemical vapour deposition technique at temperatures as low as 150°C using gallium as the catalyst. This study investigates the structure and the size of the grown silicon nano-structure as functions of growth temperature and catalyst layer thickness. Moreover, the choice of the growth temperature determines the thickness of the catalyst layer to be used.

The electrical and optical characteristics of the nano-wires were tested by incorporating them in photovoltaic solar cells, two terminal bistable memory devices and Schottky diode. With further optimisation of the growth parameters, SiNWs, grown by our method, have promising future for incorporation into high performance electronic and optical devices.

## Background

Silicon nano-wires (SiNWs) have attracted the attention of many researchers due to their structural, optical, electrical and thermoelectric properties. They are expected to be important building blocks in the future nano-electronic and photonic devices including solar cells, field-effect transistors, memory devices and chemical and biomedical sensors. Owing to their compatibility with the Si-base technology, SiNWs can be used not only as the functional units of the devices but also as the interconnects [1-6].

Various methods have been reported for SiNW fabrication, including both bottom-up and top-down techniques. Bottom-up growth methods include laser ablation, evaporation, solution-based methods and chemical vapour deposition (CVD). The CVD growth usually takes place via vapour-liquid-solid (VLS) route [7]. Many catalyst materials, mainly metals including Au, Al, Ni, Fe and Ag, have been used for the SiNW growth [1,8]. Among these metals, Au as catalyst has been the most popular and most widely investigated due to its chemical inertness and low eutectic temperature of Au-Si system. However, Au introduces deep impurity levels in Si bandgap and degrades the charge carrier mobility [8]. Therefore, alternative catalyst investigation is of crucial importance.

One of the important parameters when considering the nano-wire fabrication process is the growth temperature, as this can determine the variety of substrates that could be used, especially when there is a prefabricated layer of some temperature-dependent material. The nano-wire growth temperature is determined by the eutectic temperature of the catalyst-precursor alloy [9]; thus, the low-temperature growth will depend on the appropriate catalysts choice. Considering the characteristics of Ga, including the Ga/Si alloy low eutectic point of 29.774°C, wide temperature range for silicon solubility and its non-reactivity to form solid compound with silicon, Ga has been suggested as a good alternative to Au to grow SiNWs at low-temperatures. It is important to note that Ga does not act as catalyst for the decomposition of precursor gas as it does not assist the dissociation of SiH_4_ below its thermal decomposition point. Therefore, Ga acts only as a solvent, and the decomposition is achieved by plasma treatment (by the use of plasma-enhanced chemical vapour deposition (PECVD) system) [10].

In this study, Ga catalyst is used with an aim to grow SiNWs at a lowest temperature using PECVD technique. The growth temperature was varied between 100°C and 400°C. The grown nano-structures were characterised using scanning electron microscopy (SEM), Ultra Violet Visible spectroscopy (UV-Vis) and Raman spectroscopy.

Electronic memory devices play a vital role in our everyday life. In the last a few decades, major progress has been observed focusing on the miniaturisation of the memory size cell while increasing its density. However, materials and fabrication techniques are reaching their limits. Alternative materials and architecture of memories, as well as manufacturing processes, are considered. In order to achieve this, different types of memories such as polymer, phase change and resistance have been reported in the literature [11-13]. Two-terminal non-volatile is one of the most promising memory types for fulfilling the aim of combining low cost, high density and small size devices [14]. Therefore in this study, we present a two-terminal non-volatile memory based on SiNWs. The suitability and potential use of SiNWs for storage medium are investigated. The electrical behaviour of these devices was examined mainly in terms of current–voltage (*IV*) characteristics and data retention time (current-time) measurements.

Schottky diodes made of bulk materials do not dissipate heat quickly; hence, performance and lifespan of the device are reduced. Recently, one-dimensional (1D) nano-structures and their incorporation into Schottky diodes have been studied extensively. Due to their high surface-to-volume ratio and space between the nano-wires, diodes made of 1D nano-structure arrays can dissipate heat faster due to individual input from each wire. Therefore, integration of these nano-materials into the device will enhance its performance and lifespan [15]. The as-grown SiNWs fabricated in this study were also used in a Schottky diode, and the electrical behaviour of the device is analysed.

Solar cells fabricated with nano-wires have shown several advantages when compared to wafer-based solar cells; some of them include trapping of light, less reflection and enhanced bandgap tuning. Although these advantages do not compete to attain efficiency more than efficiencies reported until today, they help in obtaining same efficiency or less by reducing the quantity and quality of the material. Nano-wires deposited by our growth method can have a number of benefits due to their possible fabrication directly on cheaper substrates including steel, bricks, aluminium foil and conductive glass, thus reducing the price of the solar cells based on these structures. In this study, SiNW-based Schottky solar cells were fabricated and their performance tested.

## Methods

### SiNW growth

Silicon nano-wires were synthesised in a two-step growth process via VLS mechanism. At first, the gallium layer of various thicknesses was deposited onto soda-lime glass and Si/SiO_2_ substrates via thermal evaporation. SiO_2_ layer of 1 nm thickness was used as a barrier to prevent possible diffusion of Ga into Si. The thickness of the Ga layer was varied between 7.5 and 100 nm.

The samples were then loaded into an RF-PECVD reactor with radio frequency of 13.56 MHz and left for 4 h. Hydrogen gas was introduced into the chamber, while the substrate, coated with Ga layer, is being heated up to the growth temperature. Prior to introduction of the precursor gas, hydrogen plasma was created for 5 min in order to remove possible contamination and gallium oxide layer from the substrate. Silane (SiH_4_) was used as Si source. Gas flow rates, RF power, chamber pressure and deposition duration were process variables that have been investigated in detail and will be reported elsewhere.

### Fabrication of bistable memory device

For the fabrication of a bistable memory device, glass substrate was used. Al contacts were deposited by thermal evaporation. Two silicon nitride (Si_3_N_4_) dielectric layers of 20 nm each were deposited in a PECVD system, sandwiching SiNWs between the bottom and top electrodes. SiNWs were grown for 30 min from 100-nm Ga catalyst layer at 400°C. After the Si_3_N_4_/SiNW/Si_3_N_4_/Al/glass structure was fabricated, the second layer of Al contacts was evaporated to finalise the device. The device characteristics were tested by *I*-*V* and data retention time measurements.

### Fabrication of Schottky diode

SiNW-based Schottky diodes were fabricated by growing the SiNWs directly on glass substrate from 50 nm Ga at 400°C for 20 min with subsequent evaporation of both Al contacts on top of the nano-wires. The device characteristics were tested via *I*-*V* measurements.

### Fabrication of solar cells

During solar cell fabrication, a glass substrate covered with transparent conductive oxide (TCO) layer (the details of the layer will be reported elsewhere) was utilised. SiNWs were grown on top of this layer from 50 nm Ga at 400°C for 40 min. Nano-wires for the solar cell were grown using additional phosphine in the reaction chamber for n-type doping of the nano-wires. After the nano-wire growth Al dots were evaporated for top contact.

## Results and discussion

### Low-temperature growth of silicon nano-wires

As mentioned in the ‘Methods’ section, SiNWs were grown from various thicknesses of Ga catalyst layer at various temperatures. An interesting connection between the thickness of Ga and growth temperature was observed. As it will be demonstrated in this study, the thickness of the catalyst layer is crucial when choosing the growth temperature.

**Figure 1:**
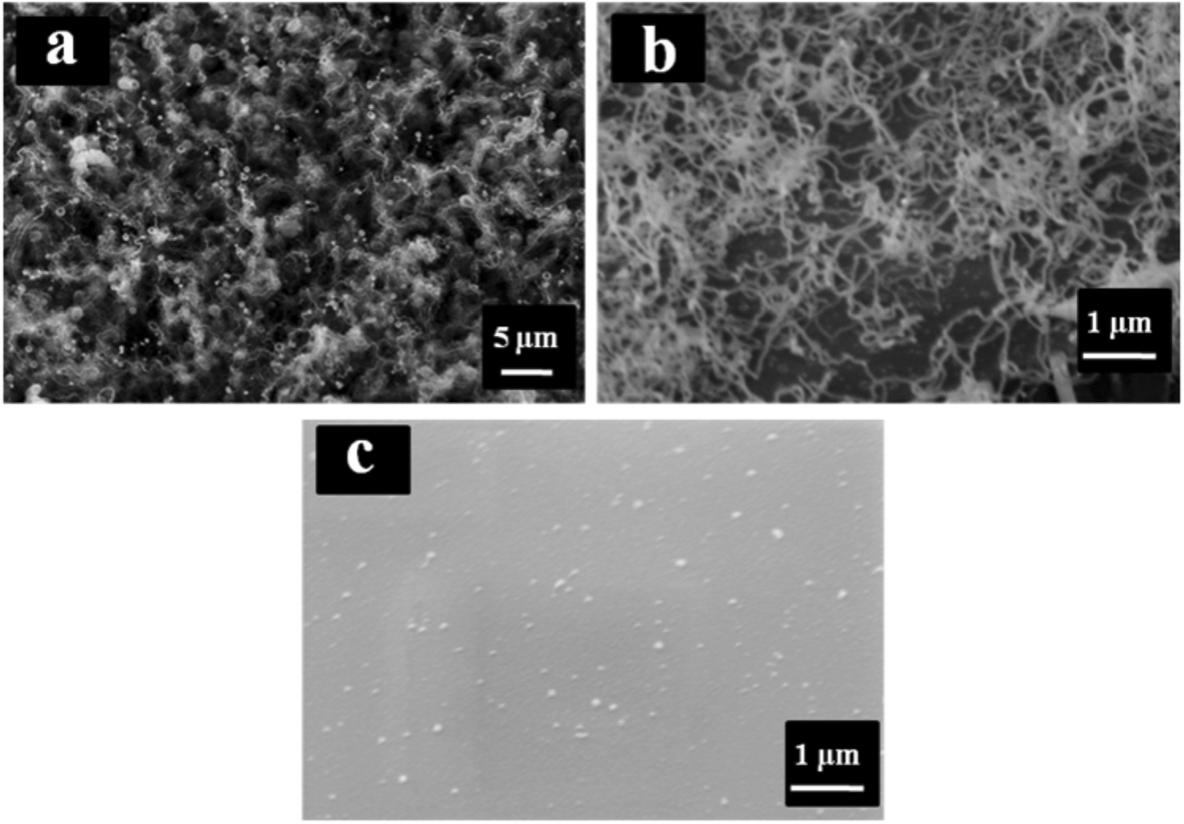
**SiNWs grown at 400°C.** (**a**) 100, (**b**) 40 and (**c**) 7.5 nm Ga catalyst layers.

SEM images of SiNWs grown at 400°C from Ga layers of 100-, 40- and 7.5-nm thicknesses are shown in Figure 1. It is noticeable that at this temperature, the growth takes place only for thicker catalyst layers, whereas there are no nano-wires observed on the 7.5-nm thick layer (Figure 1c).

**Figure 2:**
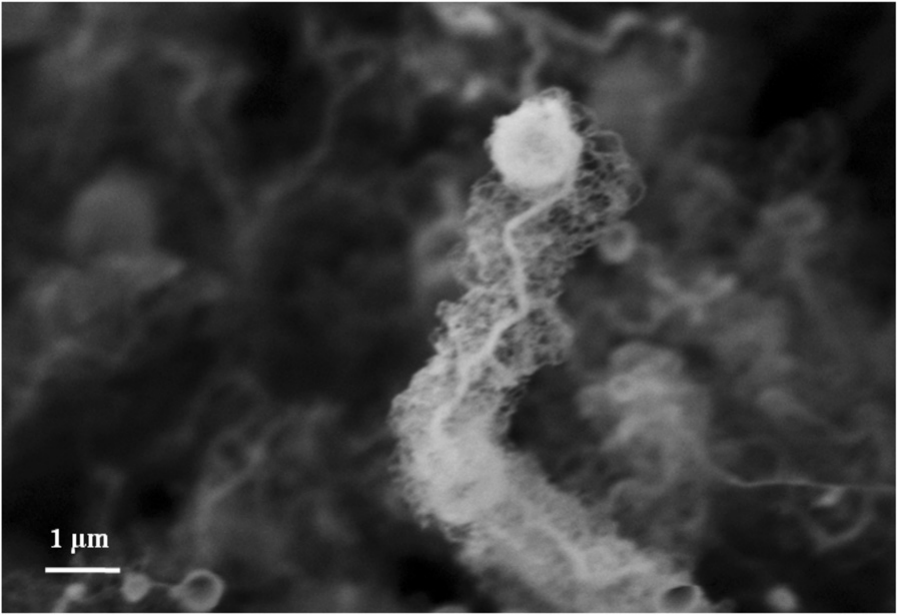
High-magnification image of the SiNWs grown at 400°C from 100 nm Ga.

The closer look at the nano-wires grown from 100-nm Ga layer (Figure 2) reveals that the growth takes place through the catalyst-at-the-top route, and the nano-wires have tree-like structures with large diameter core and thin wires grown perpendicularly from the core.

This structure was only observed for thick gallium layers at only high temperatures. A possible explanation for this is that thick layers form large Ga particles (400 nm in diameter in average for 100-nm thick Ga layer) sitting at the top of the wires which stay in a molten form at high temperatures. Therefore, the molten form of Ga slides down, covering the surface of the wire creating smaller catalyst sites for growth of thinner nano-wires from the original nano-wire surface.

**Figure 3:**
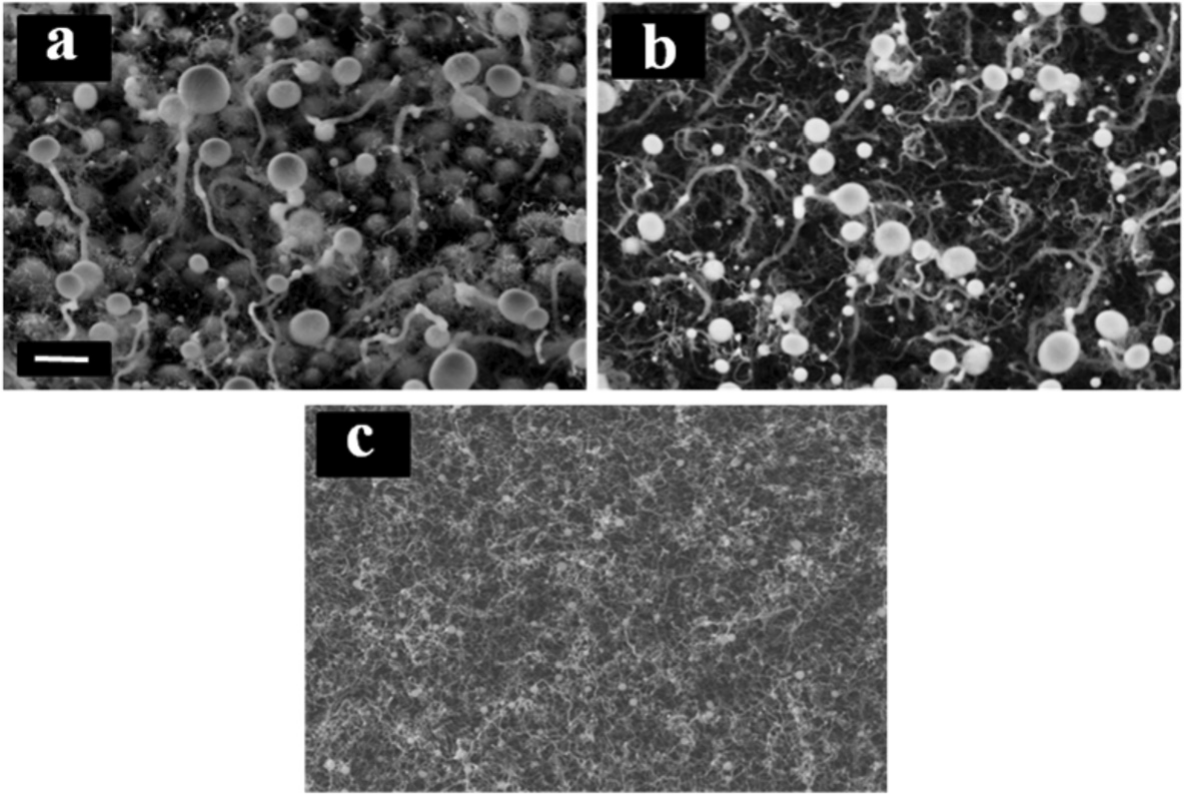
**SiNWs grown at 200°C from (a) 100, (b) 40 and (c) 7.5nm Ga catalyst layers. **The scale bar is1 μm.

Figure 3 shows SEM images of SiNWs grown at 200°C from the same thicknesses of Ga layers. It can be seen from the picture that at this temperature, nano-wire growth takes place also from 7.5-nm Ga layer, and there are no more tree-like structures formed from thicker layers.

**Figure 4:**
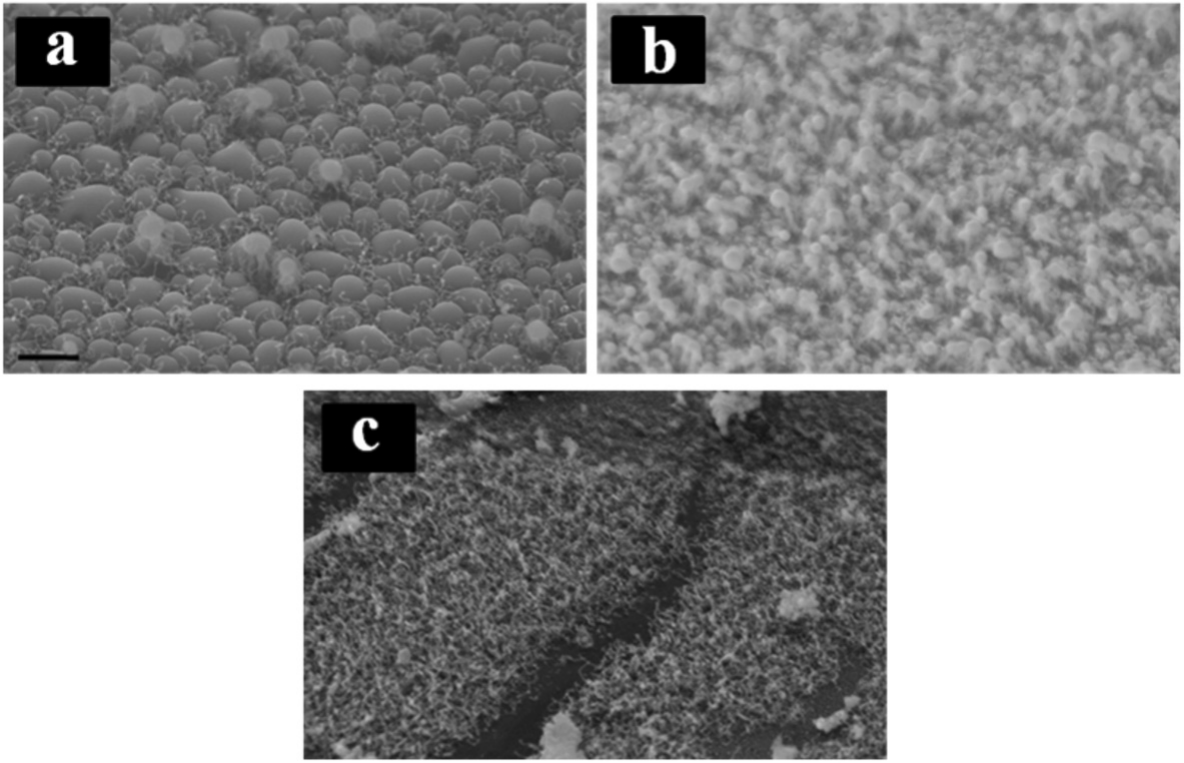
**SiNWs grown at 150°C from (a) 100, (b) 40 and (c) 7.5nm Ga catalyst layers. **The scale bar is 1 μm.

When the growth temperature was decreased down to 150°C, it can be seen from Figure 4 that only smaller catalyst particles initiate the nano-wire growth. There is no nano-wire growth observed from larger particles formed in 100-nm Ga layer (Figure 4a), but only nano-wires grown from between the big particles, possibly from smaller Ga sites that have been left at the surface of the substrate. It can be seen from Figure 4c that there are densely grown nano-wires initiated from the 7.5-nm thick Ga layer. Nano-wire growth was also observed from 40-nm Ga layer (Figure 4b).

One of the possible explanations for the abovementioned dependence of the catalyst layer/growth temperature can be the following: (a) thinner layers at high temperatures get etched away by hydrogen plasma introduced for surface pre-treatment, therefore resulting in the absence of nano-wires for these samples, (b) thicker layers create particles of larger size which at low temperatures do not reach the Si solubility level sufficient to absorb enough Si to result in supersaturation and consequent precipitation of SiNWs, whereas the smaller particles require less Si for supersaturation, therefore result in nano-wire growth.

Overall, it can be concluded that in order to grow thin diameter nano-wires using thin catalyst layers (under 10 nm), lower growth temperatures should be used, whereas thick nano-wire and tree-like nano-structure growth require thick catalyst layer and high growth temperature.

### Bistable memory device characteristics

**Figure 5:**
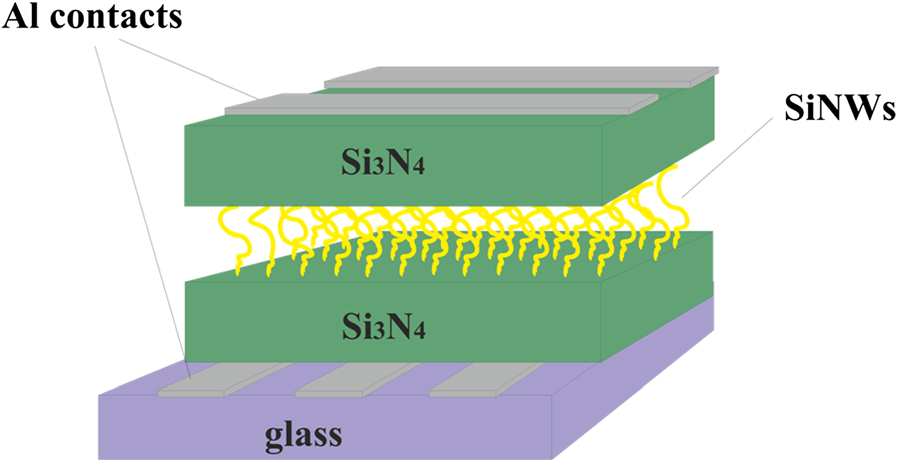
**Schematic structure of the Al/Si**_**3**_**N**_**4**_**/SiNWs/Si**_**3**_**N**_**4**_**/Al/glass bistable memory device.**

The structure of the bistable memory device fabricated in this work with SiNWs as the charge storage medium is demonstrated in Figure 5. In order to study the effect of the SiNWs in memory devices, two samples were prepared: one with SiNWs grown from Ga catalyst and the other without Ga layer referred as reference sample. Both substrates, one coated with thin layer of Ga and the other without Ga thin layer (reference sample), were placed in the PECVD chamber. The purpose of this investigation was to understand if the nano-wires grown on Ga-coated substrate had the ability to store electronic information. *I*-*V* and data retention time measurements were conducted on both samples with the aim of understanding the electronic memory behaviour.

**Figure 6:**
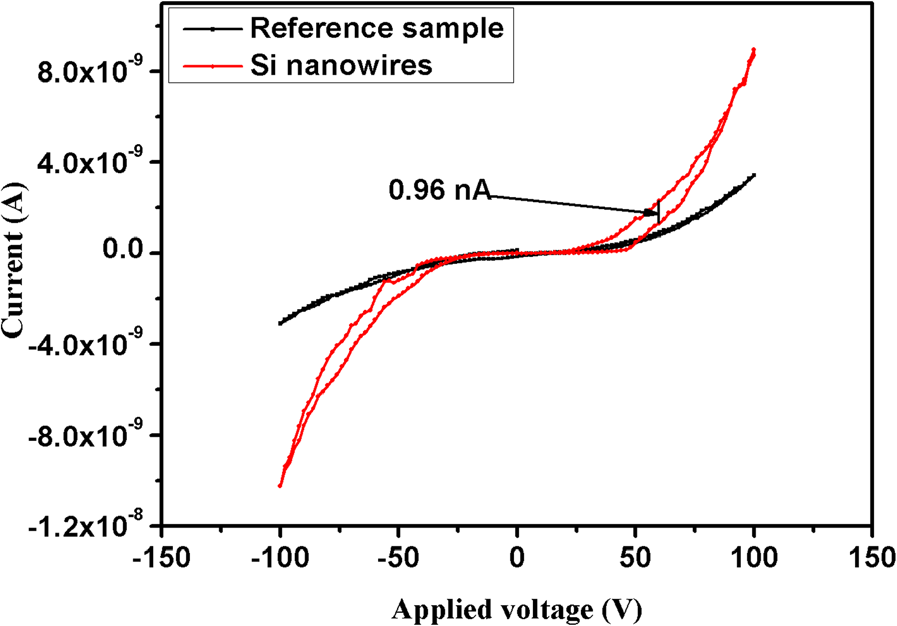
**Typical *I*-*V *characteristics of the memory cell.** The bistable memory device using SiNWs for the storage medium shows a hysteresis of 0.96 nA (red), while the reference sample (amorphous Si) shows an insignificant hysteresis (black).

Current–voltage measurements were carried out on both samples and are presented in Figure 6. It is clear from Figure 6 that the sample with SiNWs has larger hysteresis in its current–voltage behaviour as compared to the reference sample. The observed hysteresis can be attributed to the charge trapping at the interface between the layers or in the nano-wires. In this study, since there is a weaker hysteresis present for the reference sample compared to the nano-wire-based device, the charge trapping is more likely to be associated with the SiNWs. This is a strong indication that the device is able to store information. An insignificant value for charge storage was observed for the reference sample compared to that of the device with SiNWs (0.96 nA). Albeit, we are still investigating the possible explanation for the electrical bistability observed in SiNW-based devices. Here is some explanation that, we believe, causes the observed electrical bistability in our devices: when negative bias is applied on the top metal contact, electrons are injected into the SiNW structures; when a positive voltage is applied, the electrons are being extracted from SiNW structures. The presence of excess negative charge in the SiNWs may result in the observed electrical bistability. The ability to check for how long the charges could retain their state was tested by data-retention time measurements.

**Figure 7:**
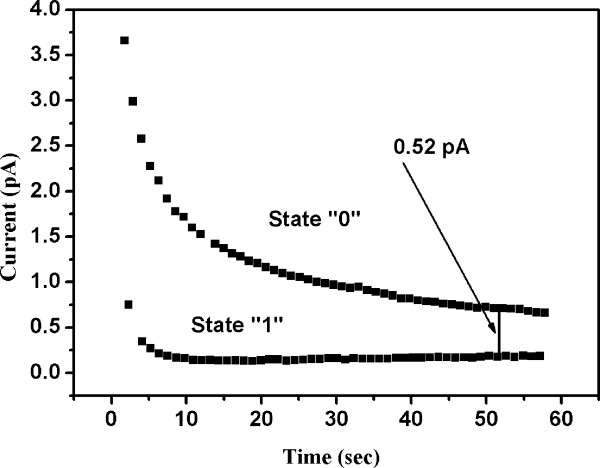
**Memory-retention time characteristics of the bistable memory device for 50 pulses.** Two different and stable electrical conductivity states (‘0’ and ‘1’) with the difference of 0.52 pA are observed.

Figure 7 shows the electrical bistability of the device by conducting data retention time measurements for 50 pulses. Firstly, a high positive voltage (100 V) is applied to the device followed by a relatively small read voltage (5 V). In that case, the device is switched to a low electrical conductivity state, referred to as the "1" state. When a high negative voltage (−100 V) is applied, the state switched to high conductivity, referred to as the "0" state.

After the initial charge loss, the two conductivity states were remained distinctive and stable as shown in Figure 7. These two states indicate that the device behaves as a non-volatile bistable memory.

### Schottky diode characteristics

**Figure 8:**
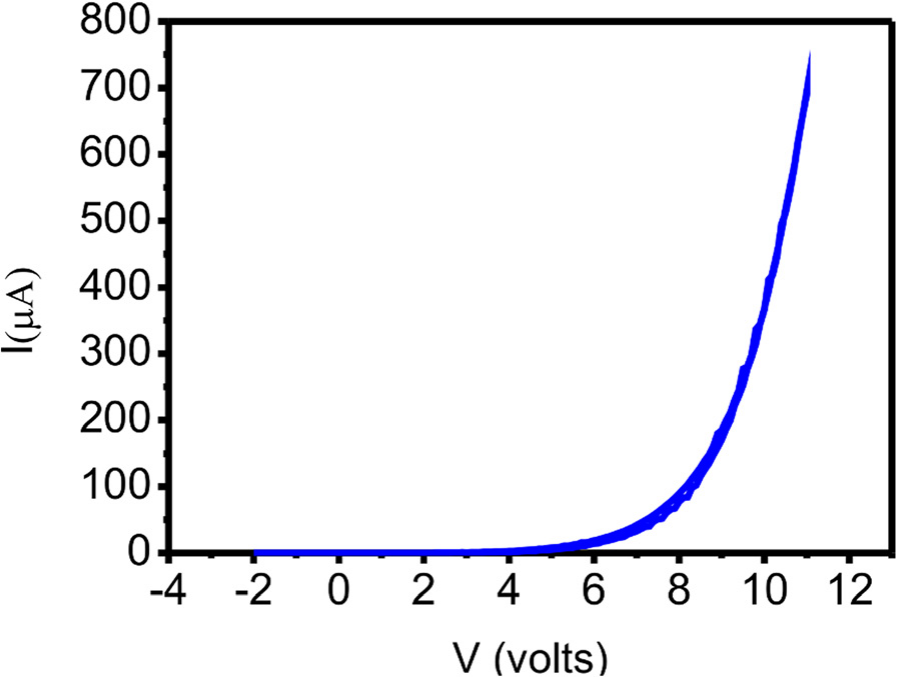
***I*-*V *characteristics of the Schottky diode with SiNWs.**

Figure 8 shows the *IV* characteristics of the Schottky junction. The response shows an exponential dependence of the current on the applied voltage, indicating that the contact is non-ohmic and it has rectifying behaviour. This is a result of Schottky barrier formation at the junction of Al and SiNWs. The formation of the Schottky barrier between the SiNWs and Al has been reported previously and is due to the large difference in work functions of these materials [16-19]. It is also observed from Figure 8 that the threshold voltage is very high, and the typical value is around 6 V (± 0.4 V). It is assumed that the electric current in Schottky contact is because of thermionic emission. The ideality factor (*n*) was estimated using the current–voltage relationship *I* = *I*_s_exp (*eV*/*nkT*) for the Schottky diode, where *I*_s_ is the reverse saturation current, *V* is the applied voltage, *k* is Boltzmann constant and *T* is the temperature in Kelvin. Ideality factor is extracted from the slope of the linear region in forward bias, and *I*_s_ is obtained by extrapolating the intercept with axis where voltage is zero from ln(*I*) vs. *V* plot. Values of *n* and *I*_s_ are obtained to be 17.68 and 91.82 pA, respectively. the high value of ideality factor may be attributed to the presence of native oxide on electrodes and non-homogenous barrier [20,21]. Some more possible reasons could be space-charge limited conduction, parasitic rectifying junctions within the device [22] and the presence of large number of surface states [23]. Further investigation is underway to unfurl this experimental observation.

### Solar cell characteristics

**Figure 9:**
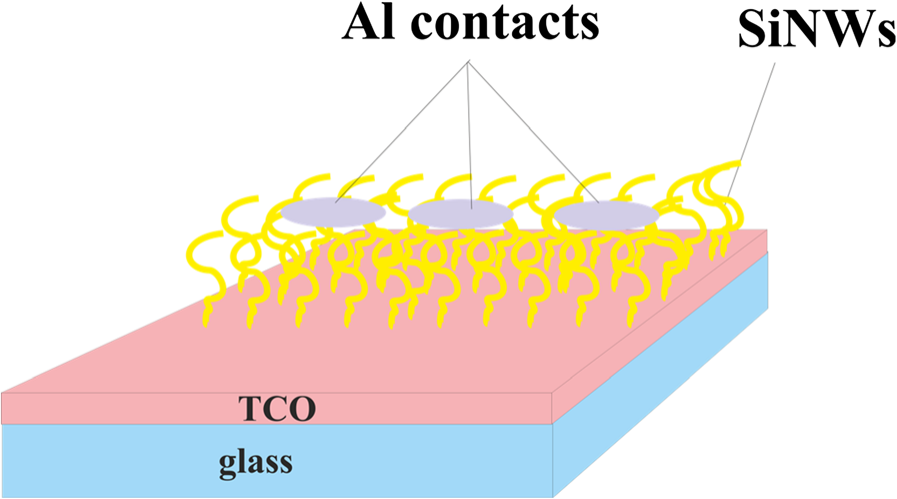
Schematic structure of the Al/SiNWs/TCO/glass solar cell.

**Figure 10:**
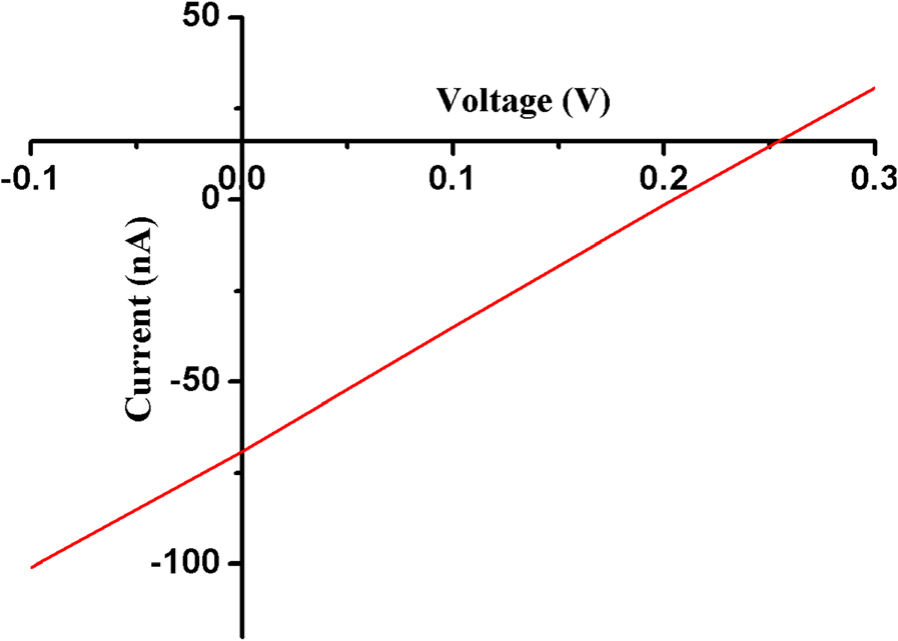
**Illuminated *I*-*V *characteristics of fabricated Schottky solar cell depicting *V***_**oc **_**and *I***_**sc**_**.**

The schematic structure of the Schottky solar cells with the Al/SiNWs/TCO/glass structure can be seen in Figure 9. Fabricated solar cell showed photoconductivity and photovoltaic characteristics. The *I*-*V* characteristics of the fabricated solar cell are shown in Figure 10. Open-circuit voltage (*V*_oc_) and short-circuit current (*I*_sc_) are measured to be 0.204 V and 70 nA, respectively, with fill factor of 0.23. The small fill factor and efficiency could be due to some parasitic resistances which actually reduce the squareness of the curve in the fourth quadrant.

The curve in the bottom right quadrant is flat, which indicates high sheet and low shunt resistances. Shunt resistance is generally caused by leakage current which arises from pinholes and recombination traps in the active layer [24]. It is reported that the leakage can also occur due to the shunting of surface leakage along with junction leakage [24]. It has been reported that silicon structures grown by PECVD process usually contain bonding defects, interstitial atomic and molecular hydrogen, some voids which actually affect the activity of photo-generation of carriers [25].

**Figure 11:**
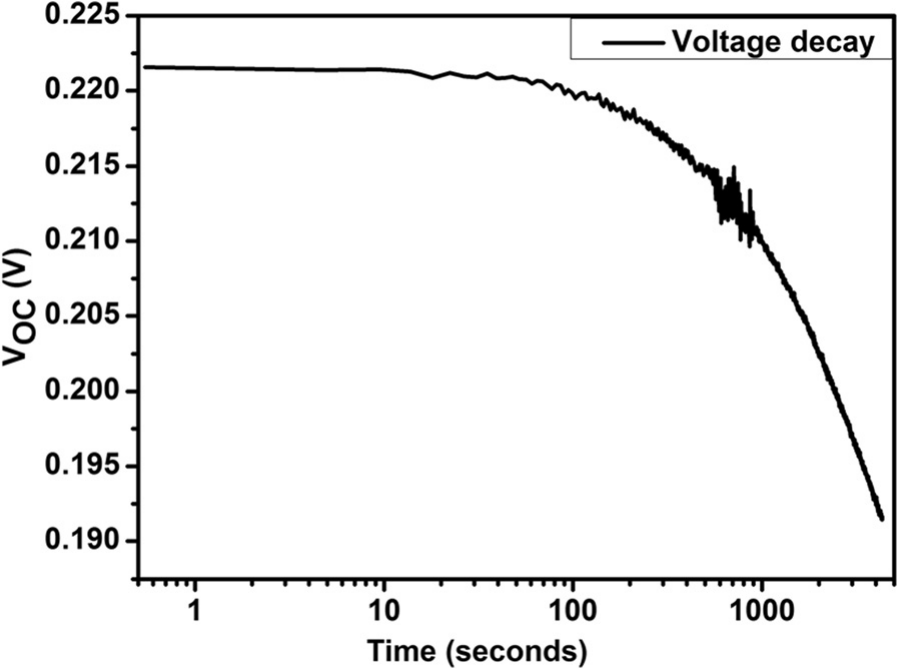
Semi-logarithmic graph of open circuit voltage of the solar cell in time.

Interestingly, the stability of the *V*_oc_ with time shows negligible change (Figure 11). Figure 11 shows semi-logarithmic graph of *V*_oc_ with time, and the change in *V*_oc_ during 1 h is measured to be 0.03 V. This change is due to the increase in temperature which actually reduces the bandgap of the semiconductor; thereby, less energy is required to break the bond, and *I*_sc_ of solar cell increases and *V*_oc_ decreases. Another parameter which strongly depends on temperature is carrier concentration of silicon which increases at higher temperatures, thereby causing decrease in open-circuit voltage [22]. The efficiency of the solar cell based on SiNWs is possible to enhance by optimising the nano-wire growth and doping, enhancing light absorption, reducing sheet resistance and modifying the surface to minimise carrier recombination as well as solar cell fabrication steps. Albeit, the photovoltaic solar cells fabricated in this study do not show high efficiency, but they do prove the point that the materials developed using the aforementioned low temperature method has wider applications. The work is currently on to improve the efficiency of the solar cell.

## Conclusions

The lowest temperature (150°C) for the growth of SiNWs via VLS mechanism is reported for the first time in literature. The growth was performed in the PECVD reactor using Ga catalyst layer. It was observed that the thickness of the Ga layer directly influences the choice of the growth temperature to be used for the nano-wire/nano-tree fabrication. The influence can be explained in two points: (a) high temperatures result in nano-tree growth from thicker layers (100 nm) of Ga, whereas thin Ga layers result in the absence of wires, (b) only thin catalyst layers (7.5 nm) initiate the growth of nano-wire arrays at low temperatures, whereas the only nano-wire growth observed from thicker layers was from between the larger particles from possible small Ga sites available.

A hysteresis of 0.96 nA was observed by the *I*-*V* characteristics of the bistable memory confirming the presence of charge trap carriers in the SiNWs. Furthermore, we detected the formation of two distinct conductivity states: a high (0) and a low (1), verifying the bistable behaviour of our memory. Schottky diode showed good rectifying behaviour with ideality factor of 17.68 and very low saturation current of 91.82 pA. Successful demonstration of silicon nano-structures to be used for Schottky diodes is shown in this paper. Though efficiency is low, silicon nano-structures play important role in light absorption which can be used as active layer for solar cells, demonstrated in this paper. Additionally, good stability of *V*_oc_ over time is also observed in solar cells. The SiNW-based bistable memory device, Schottky diode and solar cell showed promising characteristics that could be optimised further for future applications in high performance electronic and electrical energy generation devices.

## Competing interest

The authors declare that they have no competing interests.

## Authors’ contributions

NG carried out the SiNW growth for the devices and optimization of the growth conditions experimentation, and drafted the manuscript. KS carried out the bistable memory experimentation and analysis. KNM carried out the solar cell and Schottky diode experimentation and analysis. SP conceived the low temperature deposition of SiNWs idea and their exploitation into devices. He supervised the work and reviewed the manuscript. All authors read and approved the final manuscript.

## Authors’ information

NG received her BS and MSc degrees in Physics from Yerevan State University, Armenia in 2006 and 2008, correspondingly. Currently, she is a Ph.D. student at Emerging Technologies Research Centre (EMTERC), De Montfort University, investigating fabrication of nanomaterials for biosensor application. KS received her BS degree in physics at Patras University, Greece in 2010 and her MSc degree in 2011 in Microelectronics and Nanotechnology at EMTERC, De Montfort University. Currently, she is a Ph.D. student at EMETRC, De Montfort University looking into fabrication of flash memory devices on plastic. KNM received his BS degree in Electronics and Communication from Visvesvaraya Technological University, India in 2010, and his MSc degree in 2012 in Microelectronics and Nanotechnology at EMTERC, De Montfort University. Currently, he is a Ph.D. student at EMTERC, De Montfort University working on nanomaterials for photovoltaic applications. SP received his MS from the Indian Institute of Science, Bangalore, India and his Ph.D. from De Montfort University. Currently, he is the head of EMTERC, De Montfort University. He has previously worked in Cambridge University, Durham University, and Rutgers University.
